# Exploratory Profiling of Extracellular Vesicles and Citrullinated Proteins in Plasma of Greenland Shark *Somniosus microcephalus*

**DOI:** 10.3390/biology15201867

**Published:** 2026-10-16

**Authors:** Sigrun Lange, Sarah R. Needham, Igor Kraev, Benjamin M. Davis, Arve Lynghammar, Kim Præbel

**Affiliations:** 1 Pathobiology and Extracellular Vesicles Research Group, School of Life Sciences, University of Westminster, London W1W 6UW, UK; 2 Lasers for Science Facility, Central Laser Facility, UKRI: Science & Technology Facilities Council, Rutherford Appleton Laboratory, Harwell Campus, Didcot OX11 0QX, UK; sarah.needham@stfc.ac.uk (S.R.N.); benjamin.davis@stfc.ac.uk (B.M.D.); 3 Electron Microscopy Suite, Faculty of Science, Technology, Engineering and Mathematics, Open University, Milton Keynes MK7 6AA, UK; igor.kraev@open.ac.uk; 4 The Norwegian College of Fishery Science, UiT The Arctic University of Norway, NO-9037 Tromsø, Norway; arve.lynghammar@uit.no (A.L.); kim.praebel@uit.no (K.P.)

**Keywords:** Greenland shark, extracellular vesicles, citrullination/deimination, immunity, gene regulation, metabolism, homeostasis, cell senescence, oxidative stress

## Abstract

The Greenland shark *Somniosus microcephalus* is the longest-living vertebrate on Earth. With an estimated lifespan of up to at least 272 years, slow metabolism and resistance to high pressure and cold temperature, its unusual physiology remains poorly understood. Protein citrullination (deimination) is a post-translational modification of arginine into citrulline, catalysed by peptidylarginine deiminases (PADs) in a calcium-dependent manner. Citrullination of proteins changes charge, conformation and downstream protein folding, affecting protein function and protein–protein interactions. Citrullination is also linked to extracellular vesicle (EV) communication, which is crucial in cell–cell interactions. This explorative study focussed on profiling F95-enriched pan-citrullinated proteins from plasma and identifying plasma EVs from two archived Greenland shark plasma samples (from one shark sampled in 2010, storage at −20 °C, and one pool of three sharks sampled in 2015, storage at −80 °C). EVs were labelled for EV markers and citrullinated proteins were confirmed to co-localise to EVs by single-molecule detection using super-resolution microscopy. Total EV-associated proteins from the EV-enriched fraction were identified by LC-MS/MS, revealing 76 (plus seven uncharacterised) proteins associated with several immune, oxidative stress, DNA replication, cell senescence and metabolic pathways, using pathway annotation analysis (10 KEGG, 37 Reactome and 30 Biological Process GO pathways). Furthermore, LC-MS/MS analysis of F95-enriched (pan-citrullinated) proteins from Greenland shark plasma identified 75 protein hits (plus six uncharacterised proteins), which were associated with nine KEGG, 38 Reactome and 25 Biological Process GO pathway annotations linked to gene regulation, stress and immune responses, chemical homeostasis, metabolism and cell signalling. As the Greenland shark is an extremely long-living species which also tolerates cold temperatures and high pressures, understanding of EV-mediated processes and target proteins of citrullination in this species may provide insights into its unusual physiological adaptations.

## Simple Summary

The Greenland shark *Somniosus microcephalus* is the longest-living vertebrate on Earth with unusual metabolic and physiological adaptations and an underexplored model species for investigating the biology of longevity and resistance to ageing. To gain new insights into cellular mechanisms in this species, we used two archived samples from shark plasma to assess the profiles of extracellular vesicles (EVs), which are small membrane particles which carry cargoes, including proteins, between cells as a key mechanism in cell communication. We also explored candidate citrullinated proteins in shark plasma, as post-translational citrullination can modify protein function in physiological and disease pathways. EV protein content was associated with key immune, oxidative stress, DNA replication, cell senescence and metabolic pathways. Citrullinated protein candidates were associated with pathways relating to gene regulation, stress and immune responses, chemical homeostasis, metabolism and cell signalling. This is the first study to our knowledge to characterise plasma EVs in Greenland shark, including by using super-resolution microscopy, showing citrullinated protein detection co-localised to EVs. Our findings contribute to ongoing research on immune, metabolic and cell-communication pathways in this unique species, also of possible interest for human studies on longevity and extreme environment adaptations.

## 1. Introduction

The Greenland shark *Somniosus microcephalus* (Bloch & Schneider, 1801), a highly migratory species [1], is the longest-living vertebrate on Earth, with an estimated lifespan of up to 400 years [2]. It is among the largest and most widely distributed fish species throughout deep and/or cold waters of the North Atlantic Ocean, enduring high pressures at depths approaching 3000 metres and subzero temperatures as low as −1.1 °C [3,4]. *Somniosus microcephalus* has a slow metabolism and growth, and large pup sizes, while it also has an unusually high fecundity (200–324 pups per pregnancy) for an elasmobranch [5], making it vulnerable to increased fishing pressure in a warming Arctic environment [3]. *S. microcephalus* has various metabolic adaptations, including high levels of trimethylamine N-oxide (TMAO), which has roles in osmotic regulation and may depress the freezing point of body fluids, also protecting proteins against destabilising forces such as the high urea concentrations present in elasmobranchs [6]. Furthermore, diacylglyceryl ethers and triacylglycerols, together with other lipids, are thought to act as buoyancy-regulating molecules in shark livers [3]. Shark immunity is overall well developed with respect to innate and adaptive immunity [7]. The complement system has been studied in several shark species for alternative and classical pathway functions [8,9], and several immunoglobulin isotypes, including secreted forms, are reported in shark [10]. High lysozyme activity levels found in the Leydig organ, spleen and pancreas of *S. microcephalus* also suggest a well-developed immune response against various pathogens [11]. Furthermore, the haemoglobins of this polar shark have evolved adaptive decreases in O_2_ affinity in comparison to temperate sharks [12]. The extreme longevity, negligible senescence and unusual biological characteristics that could influence multiple hallmarks of ageing, including genomic stability, proteostasis and intercellular communication, have suggested the Greenland shark as a species of interest for human longevity studies [13,14].

Protein citrullination (deimination) is a post-translational modification of arginine into citrulline, catalysed by peptidylarginine deiminases (PADs) in a calcium-dependent manner. Citrullination of proteins changes their charge, increases hydrophobicity and affects protein folding, with downstream consequences for function and protein–protein interactions [15,16]. Citrullination can also contribute to neo-epitope formation of proteins, which is associated with autoimmune and inflammatory diseases [16,17]. Post-translational citrullination may furthermore facilitate protein moonlighting, which is the ability of proteins to take on multiple functions in physiological and pathobiological processes [18,19]. Protein structures most prone to citrullination are disordered proteins and beta-sheets [20]. A wide range of proteins are associated with citrullination, including cytoskeletal, mitochondrial and nuclear proteins. Citrullination of histones is involved in gene regulation, with histone H3 citrullination further implicated in neutrophil extracellular trap formation (NETosis), forming part of immune responses [21,22]. The PADs are a family of five isozymes in humans (PAD1, 2, 3, 4 and 6), with tissue-specific distribution and varying preferences for target proteins. PAD2 is the most ubiquitous PAD isozyme and is conserved across phylogeny [15]. In birds and reptiles, three PAD isozymes are found, while in fish there is one PAD (PAD2-like) form [23,24,25], including in shark [26], while PAD homologues (arginine deiminases, ADIs) are reported in bacteria and parasites [27,28,29]. Citrullination of cytoskeletal proteins has been associated with extracellular vesicle (EV) biogenesis [30,31] which forms part of cell–cell communication in health and disease. EVs are released from and taken up by cells as part of cellular communication and carry various cargoes, including proteins, lipids and RNA species [32,33]. The use of EVs as biomarkers has received increasing attention, as they can readily be isolated from various body fluids, including plasma. Recent studies have profiled EVs across a wide range of fish species, including teleost, chondrostean, cartilaginous and jawless fish [24,25,26,34,35,36,37]. The mapping of EV cargo proteomes may inform EVs’ roles in cellular communication in physiological and immune-related responses. Given the unusual physiology of the Greenland shark, understanding of EV-mediated communication, including with respect to EV-associated protein cargoes, and identification of putative citrullinated protein targets in plasma may also hold information of interest for human physiology and pathobiology.

This current explorative discovery study focussed on profiling EVs with respect to proteomic content and associated pathway annotation analysis, also confirming co-localisation of citrullinated proteins to plasma EVs using super-resolution microscopy with single-molecule detection. Candidate citrullinated protein profiles were furthermore analysed from whole shark plasma using F95 enrichment for anti-citrulline immunoprecipitation in conjunction with LC-MS/MS for identification of protein hits and associated pathway annotation analysis.

## 2. Materials and Methods

### 2.1. Sampling of Greenland Shark Plasma

**Figure 1:**
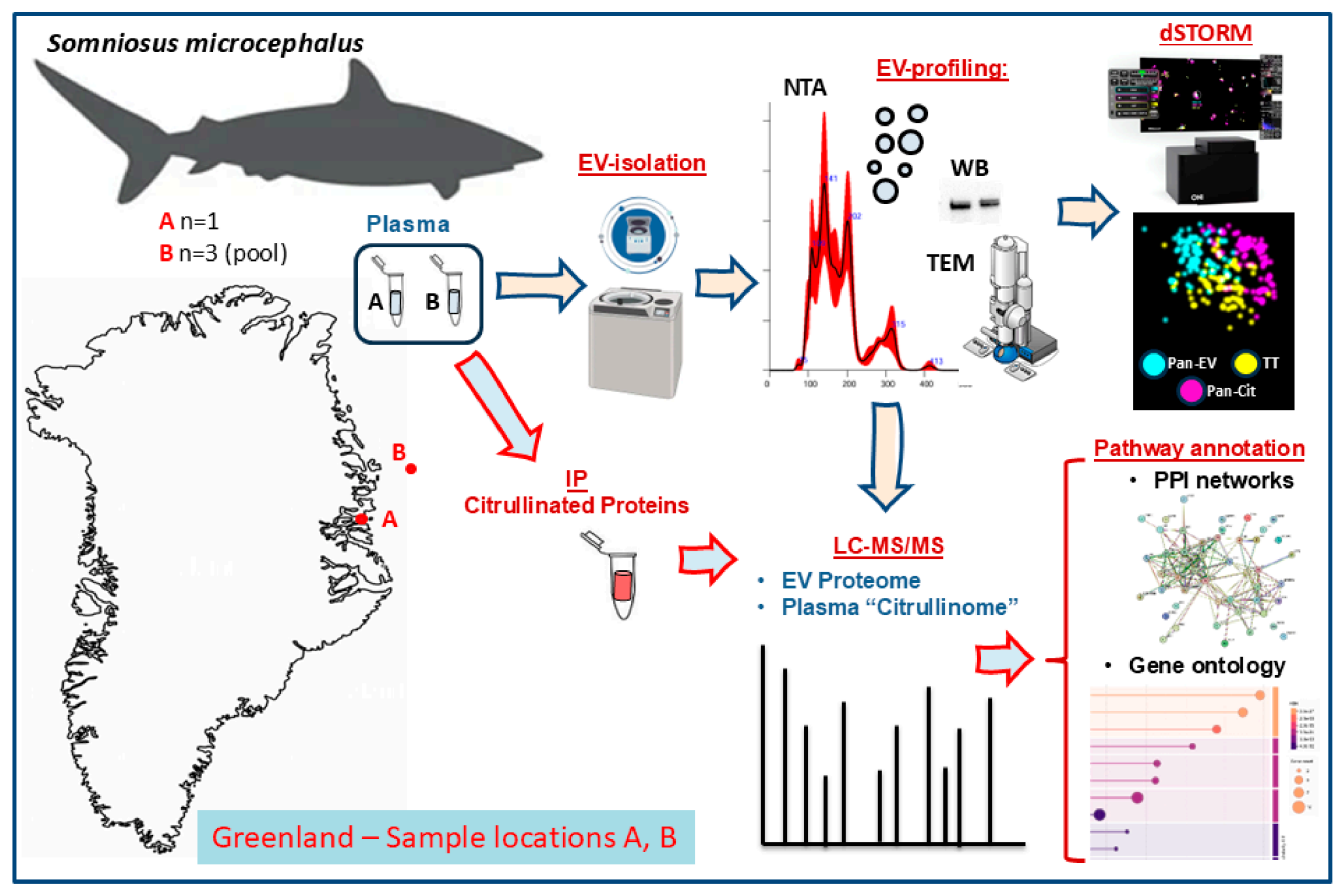
Experimental workflow. Plasma samples were used from two archived Greenland shark (*S. microcephalus*) samples (sample A from one shark; sample B a pool from 3 sharks) from the TUNU IV and TUNU VI missions, with locations indicated in the Keiser Franz Joseph Fjord (A) and the NE Greenland shelf (B) by the red circles. The two plasma samples were processed for enrichment of candidate citrullinated proteins by IP using the F95 pan-citrullination antibody and for enrichment of extracellular vesicle (EV) fractions, which were labelled by dSTORM for single-molecule detection of EV markers and citrullinated protein cargo. The EV proteome and F95-enriched plasma proteins were identified by LC-MS/MS and protein–protein interaction (PPI) networks were annotated for associated gene ontology pathways.

This study is based on two archived Greenland shark plasma samples (sample A from one individual and sample B a pool from three individuals) from the UiT, Norway. Plasma was initially collected from four Greenland sharks (*S. microcephalus*) obtained by longlining during the TUNU IV expedition (2010; expedition permit number C-10-16) and TUNU VI expedition (2015; expedition permit number C-15-17) in Northeastern Greenland (Table 1 and Figure 1). For plasma collection, blood was sampled from the caudal vein using heparinised 50 mL syringes and 16G 90 mm needles. Plasma was isolated immediately after sampling by centrifugation at 1700× *g* for 15 min and frozen; sample A was frozen at −20 °C, while sample B was pooled and frozen at −80 °C, until used for further analysis. All sampling was carried out according to Greenlandic laws and regulations. The vessel RV Helmer Hanssen must additionally comply with Norwegian laws and regulations, but no experimental animal permit was needed since the animals were sacrificed prior to sampling.

The experimental setup is shown in Figure 1, indicating the two sample locations in Greenland (A and B; red circles), plasma processing for isolation of candidate citrullinated proteins by F95-immunoprecipitation (IP) and downstream LC-MS/MS and pathway annotation analysis. Plasma EVs were enriched from the same plasma samples by differential centrifugation, followed by EV characterisation by nanoparticle tracking analysis (NTA), transmission electron microscopy (TEM) and blotting for surface markers. Direct stochastic optical reconstruction microscopy (dSTORM) was used for single-molecule detection of EV surface markers (PanEV and tetraspanins; TT) alongside detection of citrullinated EV proteins (Cit). EV proteomic content was analysed by LC-MS/MS and associated pathway annotation was carried out (Figure 1).

### 2.2. Preparation of EV-Enriched Fractions and Characterisation

#### 2.2.1. EV Enrichment by Differential Centrifugation

EV-enriched preparations were obtained from the two plasma samples by differential centrifugation using 100 µL plasma aliquots for each isolation, according to established protocols in previously published methods [25,26,38] and recommendations by MISEV2023 [33]. In brief, per EV preparation, 100 µL shark plasma was diluted 1:4 in ultrafiltered (using a 0.22 μm filter) Dulbecco’s PBS (DPBS) and centrifuged in 1.5 mL Eppendorf tubes at 4000 *g* for 30 min at 4 °C for removal of cell debris and apoptotic bodies (Microtube rotor 75003652, High Speed Multifuge X1R Centrifuge, Thermo Electron LED GmbH, Osterode am Harz, Germany). The supernatant was thereafter collected into fresh 1.5 mL Eppendorf tubes and centrifuged at maximum speed (25,500 *g*; rotor 75003652, High Speed Multifuge X1R Centrifuge; accelerator/decelerator setting 9) for 1 h at 4 °C. The supernatant was discarded and the EV-enriched preparation was resuspended in 500 μL fresh DPBS (“washing”) and spun again for 1 h at 4 °C at maximum speed as before. The supernatant wash was discarded and the enriched EVs were resuspended in 100 μL DPBS per Eppendorf tube, before further analysis by NTA, TEM, surface marker detection by Western blotting, and for EV markers and citrullinated protein co-localisation to individual EVs by dSTORM imaging as described below.

#### 2.2.2. Nanoparticle Tracking Analysis

The EV-enriched preparations from shark plasma were assessed by nanoparticle tracking analysis (NTA), based on Brownian motion of particles in suspension, using the NanoSight NS300 system equipped with a 488 nm laser and sCMOS camera (Malvern Panalytical, Ltd., Malvern, UK). The EV-enriched preparations were diluted 1/100 in DPBS before application to the NanoSight, applying 1 mL sample (10 µL EVs plus 990 µL DPBS, ultrafiltered using a 0.22 μm filter) using the automated syringe pump set at speed 50 to ensure continuous flow of the sample, with approximately 30–50 particles per frame. Camera levels were set at 10 for capture and at threshold level 5 for post-analysis processing. Videos were recorded for 4 × 60 s and replicate histograms were generated from the recordings using the NTA software (version 3.4., Malvern Panalytical Ltd.), averaging the four readings per sample to estimate particle/EV concentration (particle numbers/mL), alongside particle size distribution profiles for particle size range, particle mean and particle modal size. The NTA curves present mean and standard error for the four replicate readings per sample. Each EV-enriched sample was measured in three technical replicates of EV-enriched preparations isolated from both samples.

#### 2.2.3. Western Blotting for EV Surface Markers

Western blotting was used to assess the EV-enriched preparation for two EV surface markers, CD63 (ab68418, Abcam, Cambridge, UK; diluted 1/1000) and Flotillin-1 (ab41927, Abcam; diluted 1/1000), according to previously described methods and evidence of cross-reactivity with cartilaginous and bony fish [26,35,37,38]. Samples were prepared 1:1 in 2× Laemmli sample buffer, boiled for 5 min at 100 °C and run on 4–20% gradient TGX gels (BioRad, Watford, UK) at 165 V for 52 min, followed by Western blotting using a Trans-Blot SD semi-dry transfer cell (BioRad, UK). Membranes were blocked with 5% bovine serum albumin (BSA, Sigma, Gillingham, UK) in Tris-buffered saline (TBS) containing 0.1% Tween20 (BioRad, UK; TBS-T) for 1 h at room temperature (RT), followed by primary antibody incubation overnight at 4 °C (all diluted 1/1000 in TBS-T), washing in TBS-T and incubation at RT for 1 h with HRP-conjugated anti-rabbit IgG (1706515, BioRad, diluted 1/3000 in TBS-T). The membranes were washed in TBS-T and visualised using enhanced chemiluminescence (ECL, Amersham, UK) with digital images acquired by the UVP BioDoc-ITTM System (Thermo Fisher Scientific, Dartford, UK).

#### 2.2.4. Transmission Electron Microscopy

For visualisation of EVs by TEM, the EV-enriched preparations were resuspended in 100 mM sodium cacodylate buffer (pH 7.4) and approximately 3–5 μL of the EV suspension was placed on to a glow-discharged TEM grid with a carbon support film. The sample was partially air-dried for around 10 min, and then the grid was placed onto a drop of fixative solution, 2.5% glutaraldehyde (Agar Scientific Ltd., Stansted, UK) in 100 mM sodium cacodylate buffer (pH 7.0) for 1 min. The grid was washed in three drops of distilled water and stained with 2% aqueous Uranyl Acetate (Agar Scientific Ltd., Stansted, UK) for 1 min. Excess stain was removed with filter paper, and the grid was subsequently air-dried. The EVs were imaged using a JEOL JEM 1400 microscope (JEOL, Tokyo, Japan) operated at 80 kV, with magnifications ranging from 30,000× to 60,000×. Digital images were captured using a GATAN Rio16 digital camera (Ametek GB Limited, Leicester, UK).

### 2.3. Direct Stochastic Optical Reconstruction Microscopy (dSTORM) for Super-Resolution Imaging of EV Markers and Co-Localisation of Citrullinated Proteins to EVs

Individual shark plasma EVs from samples A and B were imaged from the EV-enriched preparations by single-molecule localisation-based super-resolution microscopy for detection of EV surface markers and co-localisation of citrullinated proteins to EVs using the EV2 Profiler 2 kit (ONI, Oxford, UK), according to methods described in [39]. The EV-enriched samples were prepared according to the manufacturer’s instructions (ONI), following the manual sample preparation protocol (EV Profiler 2), applying 10 μL of the EV-enriched samples at a particle concentration of 1 × 10^7^ to 1 × 10^8^ particles/ml. EV capture to the chip was carried out using phosphatidylserine (PS) binding, followed by fixing, permeabilisation and staining. In parallel, EV capture was also tested using tetraspanin trio (TT; CD9, CD63, CD81) binding, with the same downstream processing as for the PS capture protocol. The captured EVs were labelled with the PanEV marker (488) and tetraspanin trio (TT; CD9, CD63, CD81 (561)) antibodies supplied with the kit. In addition, the EVs were stained for citrullinated proteins (F95 pan-citrullination antibody labelled with Alexa-647 fluorophore in-house, STFC, RAL, Harwell, UK; [39]) and co-localised to EVs. The EV standard control (800-00014, ONI) provided by the kit manufacturer (ONI) served as positive control, while the negative control lane omitted application of EVs to assess unspecific background staining. dSTORM imaging buffer was applied to the chips which were immediately imaged using the Nanoimager S Mark II system with the AutoEV imaging and CODI (https://alto.codi.bio/) analysis protocol according to the manufacturer’s specifications (ONI). Laser settings were as follows for imaging: 180 mW of 488 nm laser, 44 mW of 561 laser and 170 mW of 640 nm laser. Imaging was carried out at 40 ms/frame with 4000 frames for the 488 nm laser, and 1000 frames for the 561 and 640 nm lasers, in six fields of view for each chip per sample, to stay within the duration of the 90 min span of the dSTORM buffer being active. Each chip was imaged twice, with fresh dSTORM buffer applied for the second imaging, gathering information of a total of 12 fields of view per sample. A boundary of at least 100 µm between ROIs in the first and second reads was furthermore used to avoid the risk of cross-ROI photobleaching or possible double-counting. Channel hierarchy is as follows: PanEV stain, tetraspanin trio and the user-defined custom cargo protein (in this case F95 staining). The software stack relies on automated pipelines in the CODI Cloud Platform which processes the raw dSTORM molecular localizations to extract structural particle properties (size, count, positivity, and co-localisation). For bleed-through control channel mapping calibration via NimOS was performed before acquisition, ensuring a low standard deviation of errors (<12 nm) to maintain correct spatial alignment across multi-colour channels). Post-processing photon count filtering was carried out in CODI to exclude dim, background or bleed-through noise, ensuring only true, bright localisations are counted in the designated target channel. Only STORM-validated CF dye conjugates were used to minimise spectral overlap between the channels. For quantification of positively labelled EVs, datasets (EV positivity) were recovered using the manufacturer’s recommended particle filters: particle diameter 50–500 nm, label counts ≥ 5 for the EV characterisation markers (PanEV and TT), and ≥ 2 for the protein marker of interest (F95-positive citrullinated proteins) according to previously described methods [39,40].

### 2.4. F95 Enrichment of Candidate Citrullinated/Deiminated Proteins from Greenland Shark Plasma

Total citrullinated/deiminated proteins were enriched from shark plasma samples A and B using the pan-citrullination F95 antibody (MABN328, Merck, Feltham, UK), in conjunction with the Catch-And-Release Immunoprecipitation kit (Merck), according to the manufacturer’s instructions and previously published methods using this approach cross-species, including in nurse shark [26,36,37]. In brief, 100 µL plasma was added to Sepharose columns with 10 µL F95 antibody, 10 µL Affinity Ligand and 380 µL wash buffer and incubated overnight at 4 °C on a rolling platform. The columns were washed with wash buffer to remove unbound proteins, and thereafter the F95-bound proteins were eluted with the elution buffer provided with the kit, according to the manufacturer’s instructions (Merck). The eluted F95-enriched proteins were diluted 1:1 in 2× Laemmli sample buffer (BioRad, U.K.) and either separated by SDS-PAGE (4–20% TGX gels, BioRad) for silver staining or run 0.5 cm into a 12% TGX gel by SDS-PAGE and cut out as one band for further in-gel processing for proteomic LC-MS/MS analysis carried out by Cambridge Centre for Proteomics (University of Cambridge, Cambridge, UK) as described in further detail in Section 2.6.

### 2.5. Silver Staining and Western Blotting

The enriched plasma-EV preparations and F95-enriched plasma protein preparations were diluted 1:1 in 2× Laemmli sample buffer (BioRad, U.K.) and separated on 4–20% SDS-PAGE TGX gels (BioRad) at 165 V for 55 min. Gels were either silver-stained using the BioRad Silver Stain Plus Kit (1610449, BioRad) according to the manufacturer’s instructions, or proteins were transferred to nitrocellulose membranes by Western blotting using the SemiDry Transfer system at 15 V for 1 h. Membranes were thereafter blocked in 5% BSA-TBS-T for 1 h at RT, and incubated in primary antibodies for PAD2 (ab50257, Abcam, Cambridge, UK; diluted 1/1000) for detection of PAD protein in the Greenland shark plasma (based on previous evidence for cross-reactivity in various fish, including nurse shark [26]), and for EV markers CD63 (ab68418; diluted 1/1000) and Flotillin-1 (ab41927; diluted 1/1000) for the EV-enriched preparations as described in Section 2.2.3. Secondary antibody incubation with HRP-conjugated anti-rabbit IgG (1706515, BioRad, diluted 1/3000 in TBS-T) was carried out at RT for 1 h, followed by washing in TBS-T for 5 × 10 min and visualisation using ECL (Amersham Biosciences, Buckinghamshire, UK) and the UVP BioDoc-ITTM System (Thermo Fisher Scientific, Dartford, UK).

### 2.6. Proteomic Analysis by LC-MS/MS and Associated STRING Pathway Annotation

LC-MS/MS was carried out for both the total EV proteome and for the isolated F95-enriched candidate citrullinated proteins from shark plasma for identification of protein hits. LC-MS/MS analysis was carried out following in-gel preparation of the proteins according to previously described methods [25,26,41]. In brief, proteins from each sample (EV-enriched preparations or F95-enriched protein preparations from samples A and B, respectively) were diluted in 2× Laemmli sample buffer (BioRad), loaded at 5 μg/well and run 0.5 cm into a 12% TGX gel (BioRad), and thereafter each sample was cut out as one gel band for in-gel digestion for LC-MS/MS, performed by Cambridge Proteomics (University of Cambridge, Cambridge, UK) as follows: First, the 1D gel bands were cut into 1 mm^2^ pieces, destained, reduced (DTT) and alkylated (iodoacetamide) and subjected to enzymatic digestion with trypsin overnight at 37 °C. After digestion, the supernatant was pipetted into a sample vial and loaded onto an autosampler for automated LC-MS/MS analysis.

LC-MS/MS experiments were performed using a Dionex Ultimate 3000 RSLC nanoUPLC (Thermo Fisher Scientific Inc., Waltham, MA, USA) system and an Orbitrap Lumos mass spectrometer (Thermo Fisher Scientific Inc., Waltham, MA, USA). Peptides were loaded onto a pre-column (Thermo Scientific PepMap 100 C18, 5 μm particle size, 100 A pore size, 300 μm i.d. × 5 mm length) from the Ultimate 3000 autosampler with 0.1% formic acid for 3 min at a flow rate of 10 μL/min. After this period, the column valve was switched to allow elution of peptides from the pre-column onto the analytical column. Separation of peptides were separated by C18 reverse-phase chromatography at a flow rate of 300 nL/min and a Thermo Scientific reverse-phase nano Easy-Spray column (Thermo Scientific PepMap C18, 2 μm particle size, 100 A pore size, 75 μm i.d. × 50 cm length) at a flow rate of 300 nL/min. Peptides were loaded onto a pre-column (Thermo Scientific PepMap 100 C18, 5 μm particle size, 100 A pore size, 300 μm i.d. × 5 mm length) from the Ultimate 3000 autosampler with 0.1% formic acid for 3 min at a flow rate of 10 μL/min. After this period, the column valve was switched to allow elution of peptides from the pre-column onto the analytical column. Solvent A was water + 0.1% formic acid and solvent B was 80% acetonitrile, 20% water + 0.1% formic acid. The linear gradient employed was 2–30% B in 30 min (total LC run time was 60 min including high-organic wash step and column re-equilibration). The eluted peptides from C18 column LC eluant were sprayed into the mass spectrometer by means of an Easy-Spray source (Thermo Fisher Scientific Inc.). All m/z values of eluting peptide ions were measured in an Orbitrap mass analyser set at a resolution of 120,000 and were scanned between m/z 380–1500 Da. Data-dependent MS/MS scans (3 s cycle time) were employed to automatically isolate and fragment precursor ions and generate fragment ions by higher energy collisional-induced dissociation (HCD) (Normalised Collision Energy (NCE): 38%) in the ion routing multipole. The resolution of the Orbitrap was set to 15,000 for the measurement of fragment ions. Singly charged ions, ions with greater than seven charges and ions with unassigned charge states were excluded from being selected for MS/MS and a dynamic exclusion window of 70 s was employed.

Post-run, all MS/MS data were converted to mgf files and the files were then submitted to the Mascot search algorithm (Matrix Science, London UK, version 3.1.0) and searched in the UniProt database Greenland shark database (*Somniosus_microcephalus*_20260518 (48 sequences; 12,204 residues), but due to limited protein sequence availability the search was thereafter expanded using the UniProt Chondrichthyes database (Chondrichthyes_180526; 142,694 sequences; 65,411,590 residues) for a more comprehensive analysis. In addition, the data were searched against common contaminant sequences containing non-specific proteins such as keratins and trypsin (126 sequences). A decoy database search was selected using reverse sequences from the above databases. Trypsin was selected as the enzyme and a maximum of 2 missed cleavages were allowed. Variable modifications of oxidation (M) and deamidation/citrullination (N, Q and R) were applied as well as a fixed modification of carbamidomethyl (C). The peptide and fragment mass tolerances were set to 20 ppm and 0.1 Da, respectively. A significance threshold value of *p* < 0.05 at the peptide level and *p* < 0.01 at the protein level and a peptide cut-off score of 20 were also applied. Protein inference and protein grouping details can be found on the Matrix Science website (https://www.matrixscience.com/help/protein_inference.html).

Protein hits were thereafter assessed for protein–protein interactions using the STRING (Search Tool for the Retrieval of Interacting Genes/Proteins; https://string-db.org/; accessed on 26 June 2026) database with settings set at medium confidence, and pathway annotations were identified for KEGG, Reactome and gene ontology pathways (Biological, Molecular and Cellular GO pathways) using either the human STRING database or representative shark STRING databases (*Chiloscyllium punctatum* brownbanded bamboo shark, or *Callorhinchus milii*—Australian ghostshark) as a species-specific *S. microcephalus* database is not available in STRING. The pathway annotation images were generated in STRING using default settings, showing group terms by similarity ≥ 0.8, sorting terms by signal, as presented on the *x*-axis, with FDR presented in the legend, and exported from STRING as png images.

### 2.7. Statistical Analysis

NTA curves show readings from four recordings per sample with the median line in black and SEM presented as a red line. NTA measurements for particle number, modal and mean size were based on three technical replicates from the two biological samples (A and B). dSTORM imaging was carried out in two biological samples with two technical replicates of 6 FOVs per capture, measuring positive staining signal in a total of 12 FOVs per biological sample. Graphs were created using GraphPad Prism10. F95 enrichment of candidate citrullinated proteins and isolation of EV-enriched preparations for LC-MS/MS were carried out in two biological samples (A and B). Hits for individual ion scores in the LC-MS/MS analysis were set at >37 to indicate identity or extensive homology (*p* ≤ 0.05). Protein–protein interaction network analysis and associated pathway annotation were carried out in STRING (accessed 26 June 2026) for multiple proteins, using basic settings with medium confidence. Due to the descriptive and explorative nature of this study further statistical comparisons were not carried out. The results should therefore by interpreted as exploratory; any estimates of biological variability cannot be determined given the small sample size.

## 3. Results

### 3.1. Characterisation of EV-Enriched Preparations from Shark Plasma

**Figure 2:**
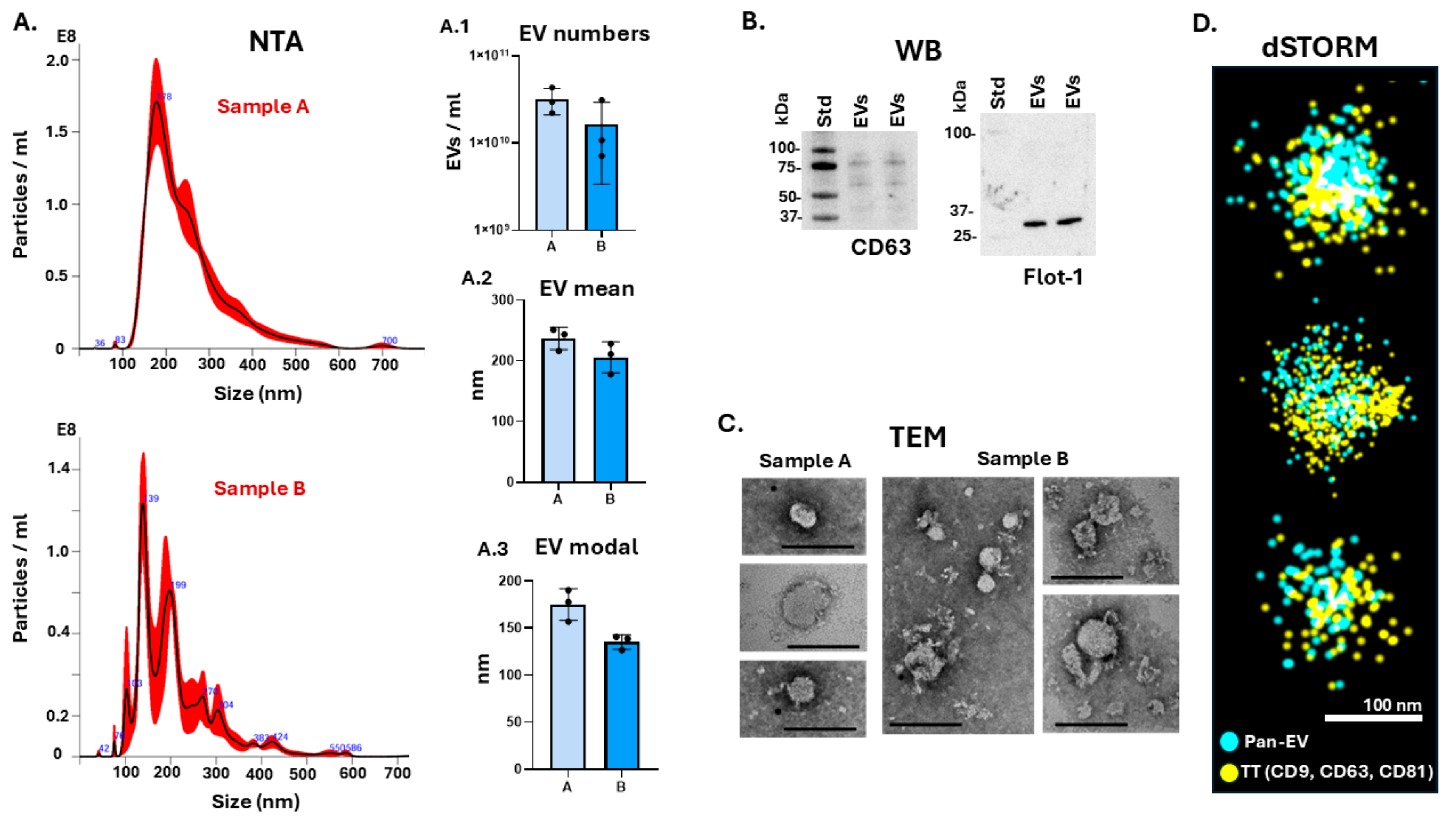
Characterisation of EV-enriched preparations from Greenland shark plasma by: (**A**) NTA, showing a representative sample curves for shark samples A and B, respectively; (**A.1**) particle numbers per ml in plasma of the two samples; (**A.2**) EV particle mean size (nm) of samples A and B; (**A.3**) EV particle modal size (nm) of samples A and B; graphs represent three technical replicates (EV isolations) from both biological samples. (**B**) Western blotting of EV surface markers CD63 and Flot-1; size standard (Std) is indicated in kDa. (**C**) TEM imaging of EV isolations from both samples (scale bars = 100 nm); (**D**) individual shark plasma EVs visualised by dSTORM single-molecule detection for PanEV marker (blue) and tetraspanin trio (TT, CD9, CD63, CD81) EV marker (scale bar = 100 nm for all images).

The EV-enriched preparations from shark plasma were analysed by NTA showing a concentration of approximately 2 × 10^10^ particles/mL, in the size range of mainly 60–350 nm, with an overall range detected up to approximately 450 nm in both samples assessed (Figure 2(A, A.1)). EV particle mean size was in the range of 178–251 nm (Figure 2(A.2)), while EV particle modal size was in the range of 126–190 nm (Figure 2(A.3)). EV surface marker detection was confirmed for CD63 at an expected size range of 30–65 kDa, and Flotillin-1 at approximately 35 kDa, respectively, by Western blotting (Figure 2B). EVs were visualised by transmission electron microscopy (TEM) with representative images shown for both samples in Figure 2C, and of individual shark plasma EVs by dSTORM imaging for single-molecule detection of tetraspanin trio marker (CD9, CD63 and CD81; TT, yellow) and PanEV marker (blue) (Figure 2D).

**Figure 3:**
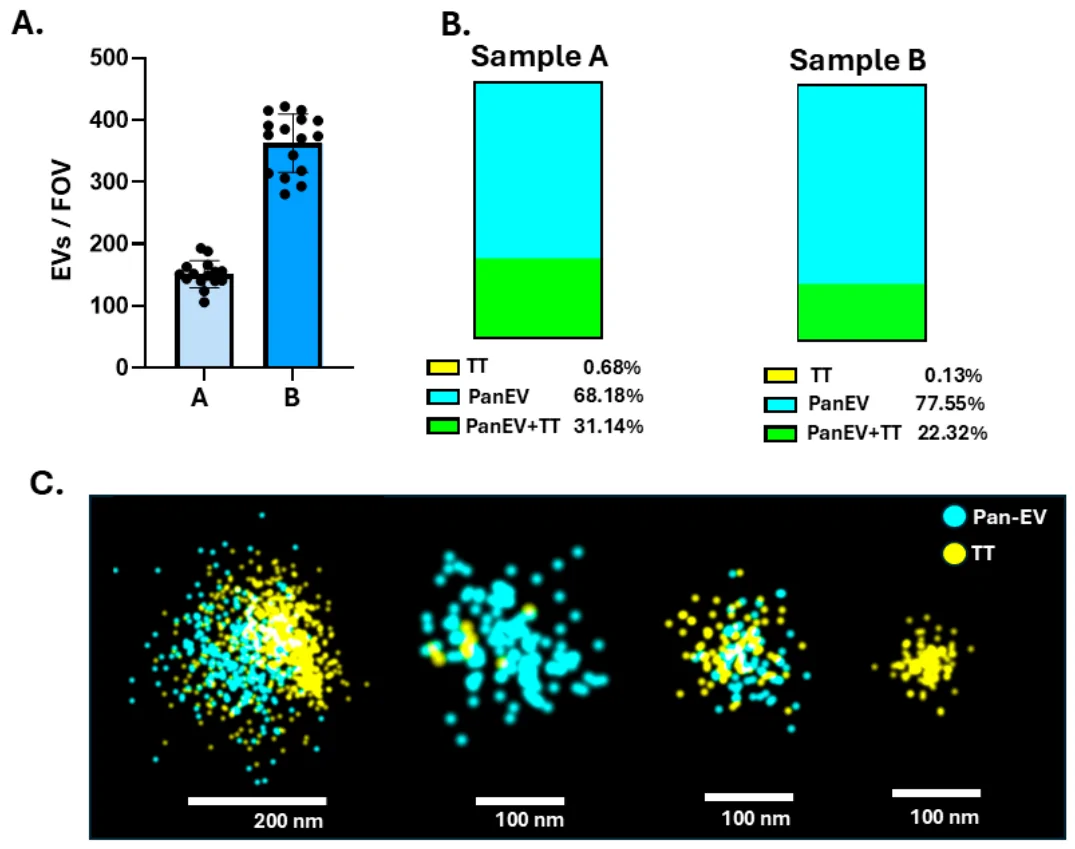
Shark plasma EVs captured by PS and labelled with EV markers for imaging and quantification by dSTORM. (**A**) Numbers of EVs captured to the ONI chip by PS binding per field of view with 12 fields of view (each presented by a black circle) assessed from the two shark plasma-EV samples. (**B**) Distribution of positive EV surface marker staining (PanEV or TT) for shark plasma EVs from both samples. (**C**) Examples of individual shark plasma EVs imaged by dSTORM for the detection of PanEV and TT markers (size bars represent 100 nm or 200 nm). EV analysis was filtered for particle size 50–500 nm.

Positive EV labelling by single-molecule detection was carried out using dSTORM, following binding of EVs by either tetraspanin trio (TT) or phosphatidyl serine (PS) capture, for the two shark samples used in this study. TT capture resulted in negligible EV binding to the chip surface, with only 3–20 particles per FOV, compared with 120–400 particles per FOV using PS binding. Hence, only the PS-bound EVs were further analysed for samples A and B. While both samples showed efficient binding of EVs to the ONI chips with the PS capture, a higher number of bound particles was observed for sample B (Figure 3A). EVs were assessed in 12 fields of view for both samples, to collect an overall estimate of positive-labelled EVs. As the dSTORM buffer life is 90 min, and recommended number of FOVs to avoid bleaching is six FOVs per sample per run, the imaging was repeated for a second time with fresh dSTORM buffer to collect information from overall 12 FOVs. For sample A, positively labelled EVs were 120–170 per field of view, and for sample B positively labelled EV numbers were 290–400 per field of view (Figure 3A). The negative control lane containing no EVs showed negligible staining, confirming absence of unspecific background staining. When assessing overall proportion of positively stained EVs for the PanEV and TT markers, a similar staining pattern was observed for samples A and B, with approximately 68–77% of EVs positive for the PanEV marker and 22–31% of EVs double-stained for PanEV and TT markers, while TT single-stained EVs were negligible (≤1%; Figure 3B). Examples of individual shark plasma EVs imaged by dSTORM using the ONI imaging system, labelled for the PanEV and TT markers, are presented in Figure 3C.

### 3.2. Co-Localisation of Citrullinated (F95-Labelled) Proteins to Shark Plasma EVs

**Figure 4:**
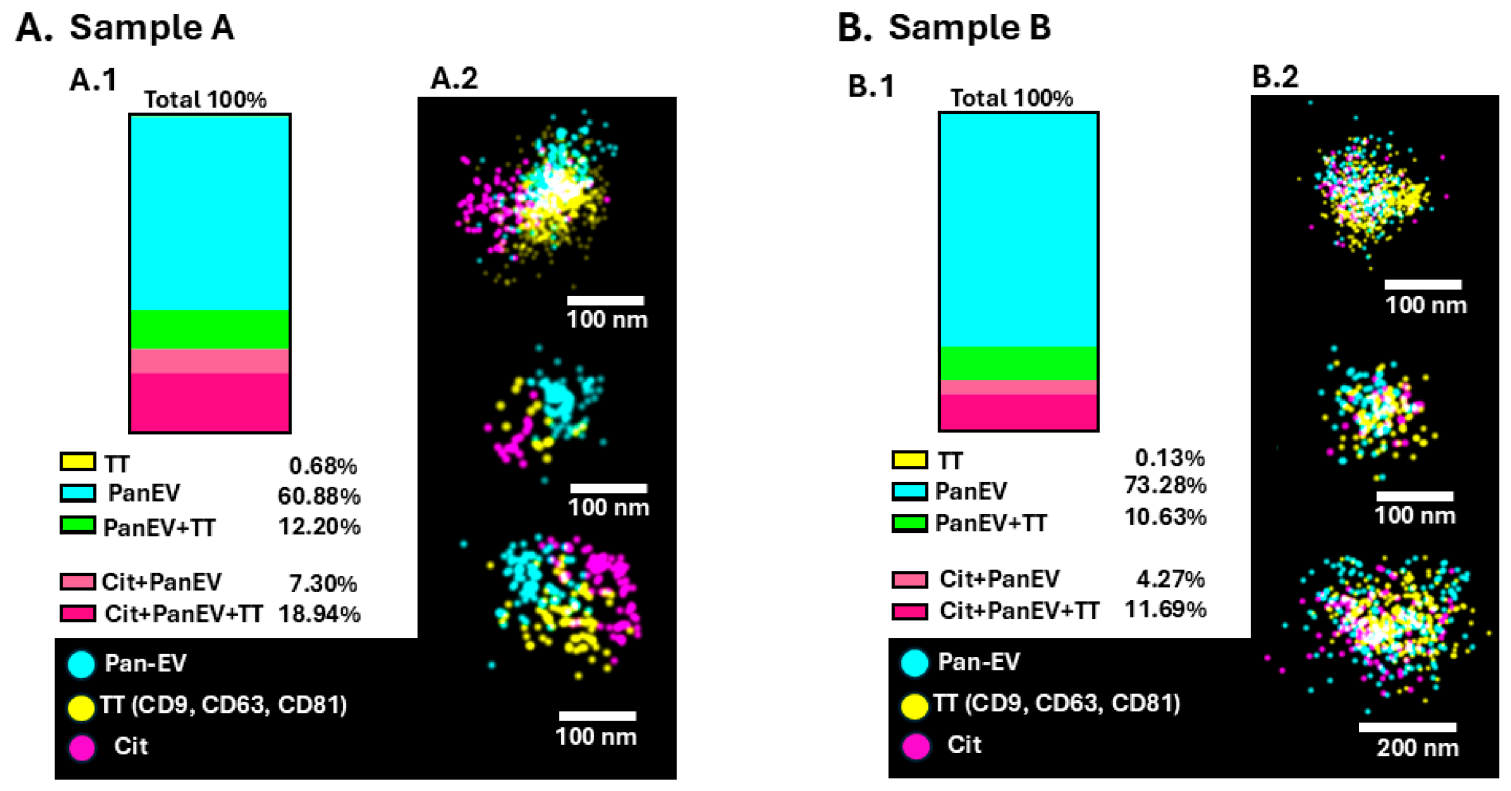
Detection of citrullinated proteins co-localised to shark plasma EVs by dSTORM. Plasma EVs from sample A (**A**) and sample B (**B**). (**A.1**,**B.1**): The column graphs show percentage positive staining for citrullinated proteins (Cit) co-localised to PanEV- and PanEV-TT-labelled EVs (*n* = 2 biological samples; *n* = 2 × 6 technical repeats for a total of 12 FOV per sample). (**A.2**,**B.2**): Examples of individual dSTORM-imaged shark plasma EVs showing citrullinated proteins (F95-positive) co-localised to PanEV-TT-positive-stained EVs in both samples; size bars are indicated (100 nm or 200 nm) and the colour key represents PanEV (blue), TT (yellow) and citrullinated proteins (magenta). EV analysis was filtered for particle size 50–500 nm.

The EV-enriched fractions were further assessed for positive staining of citrullinated proteins using the F95 pan-citrulline antibody conjugated with 641 fluorophore [39] for co-localisation to PanEV- and TT-labelled EVs. The two shark EV samples showed positive citrullination staining in the range of approximately 4.3–7.3% co-localised with PanEV-positive EVs (Figure 4(A.1,B.1)), and 11.7–18.9% citrullination positivity co-localised with PanEV-TT-positive EVs (Figure 4(A.1,B.1)). Example images showing pan-citrullination (Cit) detection co-localised to individual EVs from samples A and B are provided in the panels in Figure 4(A.2,B.2).

### 3.3. LC-MS/MS Analysis of EV-Enriched Preparations from Greenland Shark Plasma and Associated Pathway Annotation

**Figure 5:**
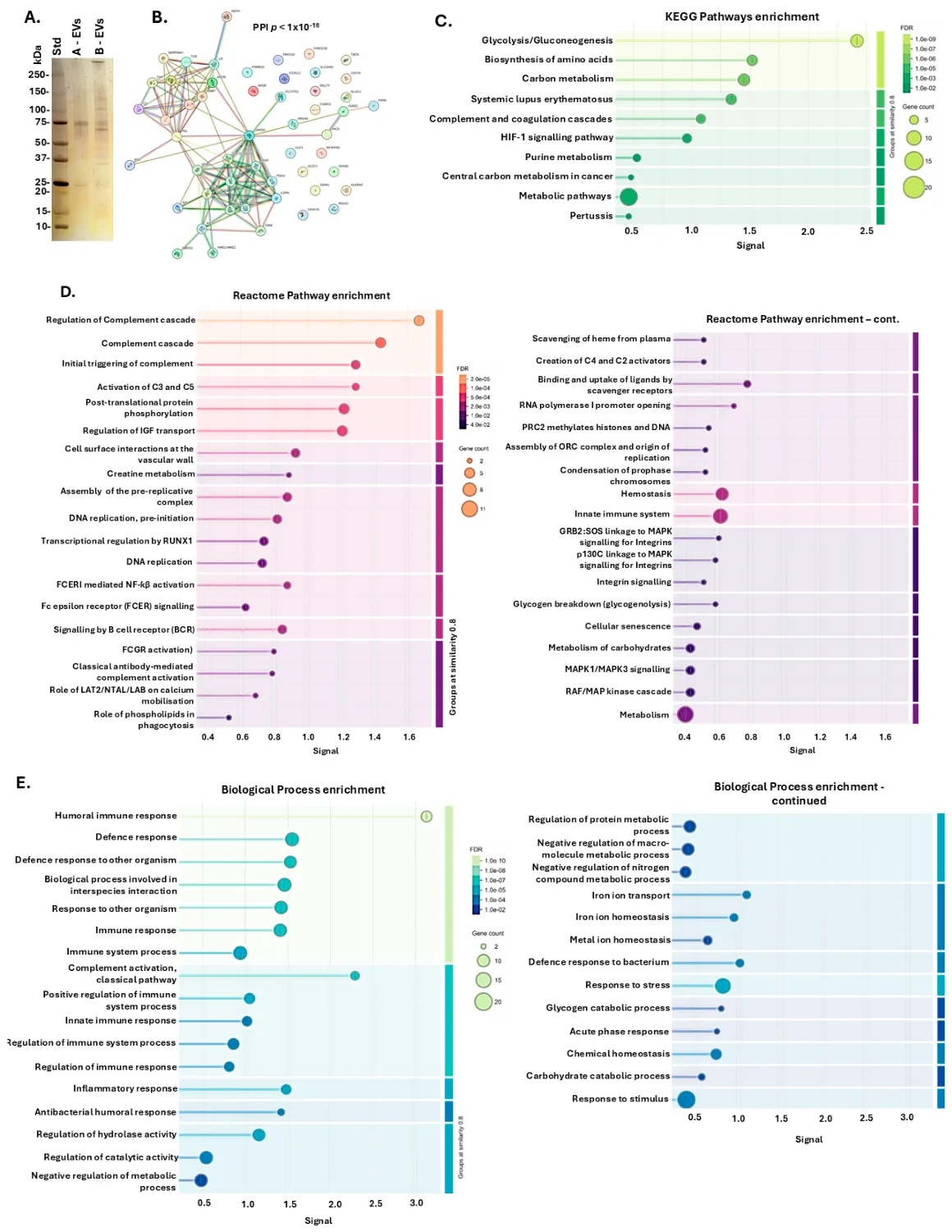
Profiling of proteins in the EV-enriched preparations from Greenland shark plasma. (**A**) Total proteins detected in the EV-enriched fractions by silver staining following SDS-PAGE (4–20% TGX gel) in samples A and B; (**B**) protein–protein interaction network analysis of the EV-associated proteins identified by LC-MS/MS, based on the human database; (**C**) KEGG pathway annotations of EV-associated proteins based on the human database; (**D**) Reactome pathway annotations of the EV-associated proteins based on bamboo shark (*Chiloscyllium punctatum)* STRING database, where 31 protein hits were identified and associated with 37 Reactome pathways; (**E**) Biological Process GO annotations of the EV-associated proteins based on 37 protein hits identified in the *Callorhinchus milii* (Australian ghostshark) STRING database, associated with 30 GO pathways. The graphs show signal on the *x*-axis; false discovery rate (FDR) is presented in the legend. Gene count indicates number of proteins associated with the term.

Protein hits identified by LC-MS/MS analysis in the EV-enriched preparations from the two shark plasma samples are listed in Table 2. Proteins detected in these EV-enriched preparations are furthermore shown by silver staining in Figure 5A. Overall, more protein hits and protein bands were observed for sample B. A total of 76 proteins and seven uncharacterised hits were identified in both EV samples combined; a summary list of all protein hits identified in the EVs by LC-MS/MS in both samples is provided in Table 2; the full LC-MS/MS analysis is provided in Supplementary Tables S1 and S2 for samples A and B, respectively.

Pathway annotation analysis was carried out using the STRING database. Protein–protein interaction network (Figure 5B) analysis for the protein hits and associated pathway annotation for KEGG (Figure 5C), Reactome (Figure 5D) and Biological GO pathways are presented (Figure 5E). To obtain the most comprehensive explorative overview of pathway annotations, the human (*Homo sapiens*) STRING database was used in the first instance based on protein hit names to generate the initial PPI network shown in Figure 5B. Overall, 10 KEGG pathway annotations were identified based on the human STRING database; these pathways were also identified to varying degrees in several different representative shark databases, as a specific Greenland shark database is not available in STRING. Using the bamboo shark (*Chiloscyllium punctatum*) database, 31 protein hits were identified in STRING, which were associated with 37 Reactome pathways for the EV proteome (Figure 5D). In addition, 37 protein hits matched with the *Callorhinchus milii* (Australian ghostshark) STRING database, which was used to generate Biological Process GO annotations, identifying 30 associated pathways (Figure 5E). This combined comparative approach allowed for the highest identification of pathway annotations associated with the EV proteome profile identified in Greenland shark plasma EVs, but in-depth interpretation requires some caution and remains explorative at this stage.

### 3.4. Proteins Enriched by F95 Anti-Citrulline Immunoprecipitation and Identified by LC-MS/MS in Greenland Shark Plasma and Associated Pathway Annotation

F95-enriched protein preparations from Greenland shark plasma were assessed for hit matches by LC-MS/MS for identification of putative citrullinated protein candidates. Overall, 75 proteins and six uncharacterised hits were identified in the two shark samples combined, as listed in Table 3; the full LC-MS/MS analysis for the F95-enriched samples is provided in Supplementary Tables S3 and S4 for samples A and B, respectively. As observed for the EV proteome analysis, more hits were identified for sample B.

**Figure 6:**
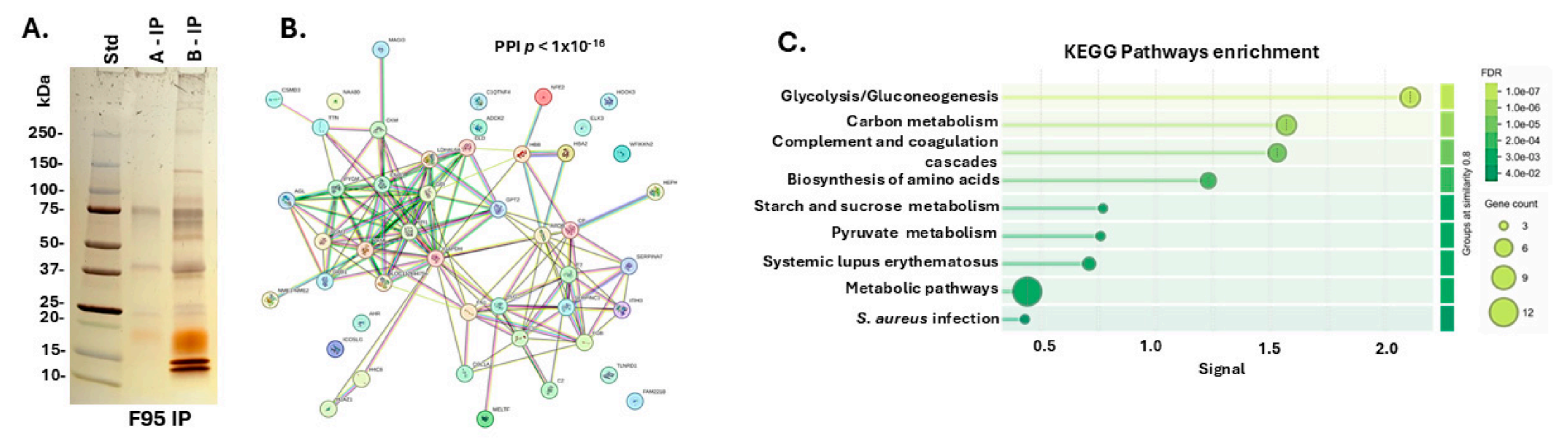

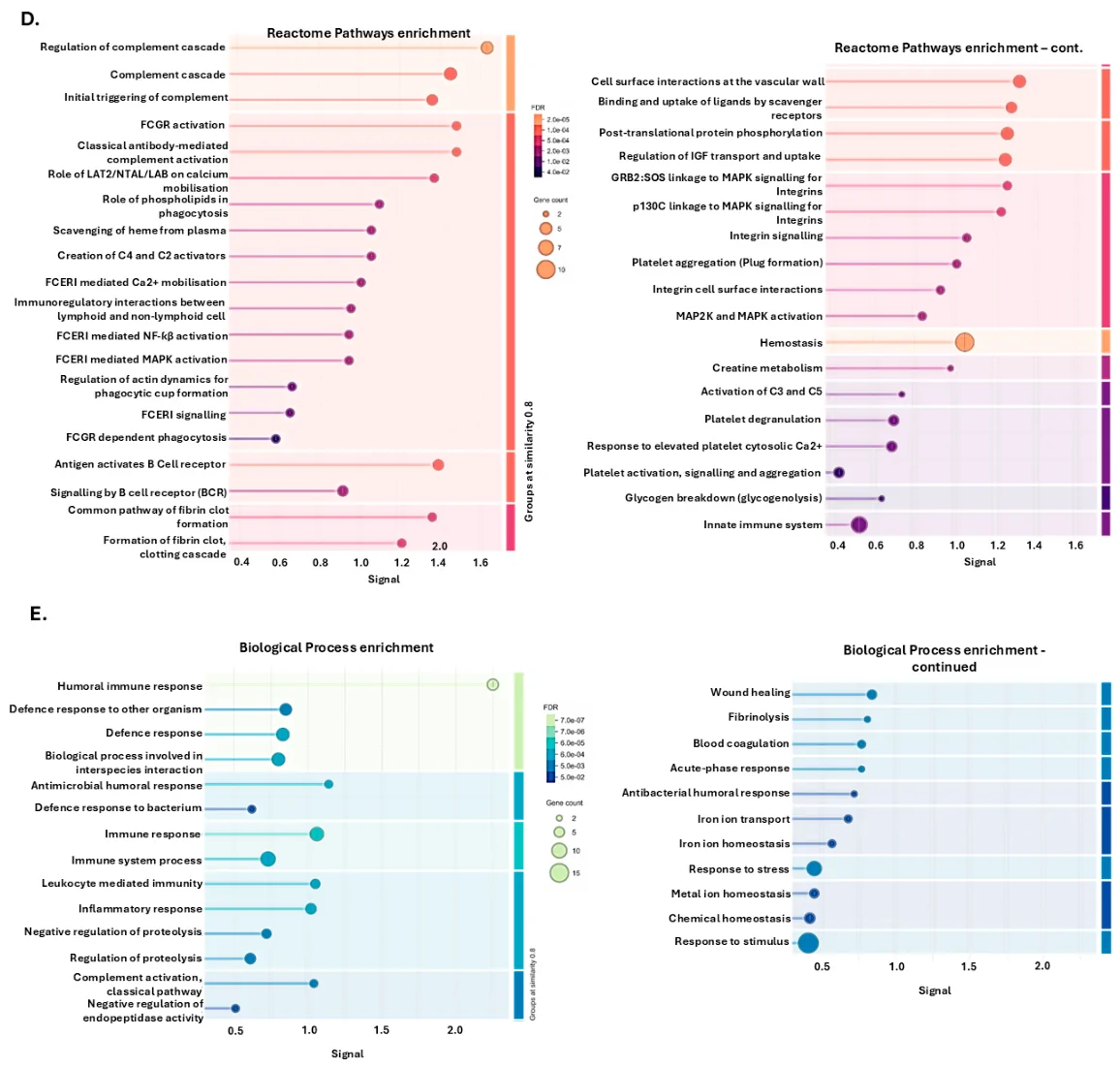
Proteins enriched by anti-citrulline (F95) immunoprecipitation from Greenland shark plasma and associated pathway annotation by STRING analysis. (**A**) F95-enriched proteins isolated from Greenland shark plasma (samples A and B) are shown by SDS-PAGE (4–20% TGX gels) and silver staining. (**B**) PPI network of F95-enriched candidate citrullinated protein hits identified by LC-MS/MS, based on protein names using the human STRING database. (**C**) KEGG pathway annotation associated with the F95-enriched protein hits in Greenland shark plasma, based on the human STRING database. (**D**) Reactome pathway annotation associated with the F95-enriched protein hits in Greenland shark plasma, based on the cloudy shark (*Scyliorhinus torazame*) STRING database, where 33 protein hits were identified. (**E**) Biological Process GO annotation associated with the F95-enriched protein hits in Greenland shark plasma, based on the brownbanded bamboo shark (*Chiloscyllium punctatum*) STRING database, where 30 protein hits were identified. The graphs show signal on the *x*-axis; false discovery rate (FDR) is presented on the *y*-axis. Gene count indicates number of proteins associated with the term.

The F95-enriched shark plasma proteins are furthermore shown by SDS-PAGE and silver staining in Figure 6A. The presence of PAD2 immunodetection was confirmed in shark plasma by Western blotting showing positive band detection in the expected 70–75 kDa size range and is shown in Supplementary Figure S1A. In addition, total protein staining of shark plasma compared with the pan-citrullination F95-enriched proteins is shown in Supplementary Figure S1B. Pathway annotation analysis was carried out in STRING for protein–protein interaction networks (Figure 6B) of the F95-enriched (candidate citrullinated) proteins from shark plasma, identifying nine KEGG pathways, based on protein names using the human STRING database (Figure 6C). When using protein IDs or names on different shark STRING databases, only a part of the protein hit list was identified, but this approach was used here for maximum identification of Reactome pathways (38 pathways), which was based on the cloudy catshark (*Scyliorhinus torazame*) STRING database, where 33 protein hits were identified (Figure 6D). Biological Process GO annotation (25 pathways were identified) for all F95-enriched protein hits that corresponded to the *Chiloscyllium punctatum* (brownbanded bamboo shark) STRING database, where 30 protein hits were identified, is presented in Figure 6E, as it showed the most hits for shark species databases available in STRING. In-depth interpretation therefore requires some caution and remains explorative only at this stage.

## 4. Discussion

This study analysed F95-enriched proteins (candidate citrullinated proteins isolated with the pan-citrullination F95 antibody) of plasma from Greenland shark (*S. microcephalus*). Profiles of plasma-EV-enriched preparations were also assessed, including with respect to total EV proteome cargoes and co-localisation of citrullinated proteins to EVs by single-molecule detection using dSTORM imaging. The two archived plasma samples used in this study were obtained from two Arctic (TUNU IV and TUNU VI) expeditions in 2010 and 2015 respectively; one was from a single shark and the second from a sample pool from three sharks. The samples had an additional variable, having been stored at −20 °C or −80 °C, respectively.

Shark plasma-EV (particle) size distribution was observed in the 60–350 nm size range, comparable with findings from nurse shark showing 40–300 nm size distribution [26] and similar to EV profiles of several whale species and orca [42]. This is to our knowledge the first study to use single-molecule detection with super-resolution microscopy (dSTORM) on shark EVs in conjunction with the commercial ONI kit, which showed successful cross-reactivity with capture and detection of shark EVs. We assessed capture of the shark EVs to the chips both by TT binding and PS binding using the commercial reagents provided with the kit, showing higher efficiency for Greenland shark plasma-EV capture with the PS capture reagent, and negligible capture with the TT capture reagent. For dSTORM analysis of the shark EVs, PanEV marker and tetraspanin trio marker (CD9, CD63 and CD81) conjugated to fluorophore were used, and the proportional labelling of the shark EVs showed that the majority of EVs were positive for PanEV staining (approximately 68–77%) followed by PanEV-TT double-staining (approximately 22–31%). The negligible detection (≤1%) of TT-only-positive EVs may correlate to the fact that EVs were bound by PS capture but may also reflect the poorer binding capacity observed for the TT capture of shark EVs to the ONI chip. Our findings indicate that PS capture is more appropriate than TT capture for cross-species application of the ONI kit’s commercial reagents (which are primarily designed for human samples), and we have observed similar trends in previous studies on mouse and crustacea biofluids [40,43]. When co-localising citrullinated protein detection to individual EVs, using the pan-citrulline F95 antibody conjugated to fluorophore, overall detection of pan-citrullinated proteins was approximately 12–18% in PanEV-TT co-labelled EVs and approximately 4–8% in PanEV-labelled EVs. This correlates to previous studies on citrullinated protein detection in several fish species using LC-MS/MS [24,25,26,34,35,37]. Our findings also correspond to the detection of pan-citrullinated proteins in human cancer-cell-derived EVs using similar dSTORM approaches on permeabilised and non-permeabilised EVs [39]. The current study assessed only permeabilised shark EVs, and hence the F95-positive detection may relate both to citrullinated proteins in the EV cargo as well as on the EV corona. In previous studies on human cancer cells, we confirmed that the majority of pan-citrullination positive signals were found in permeabilised EVs, when compared with non-permeabilised EVs, using the same ONI kit [39].

Various study limitations need to be considered. Some differences were observed between the two archived shark plasma samples analysed in this current study, both with respect to protein and EV analyses. Challenges in standardisation of EV profiling and EV purity across samples (including co-isolation of lipoproteins and possible carry-over due to haemolysis) are well acknowledged [33]. Additional challenges in archived samples, including in wild biology research but also relevant for longitudinal cohort studies, clinical samples and biobanks, are variations in sample collection, sample storage conditions and sample age, which may also affect the quality of EV profiling results [44,45]. Our findings suggest that long-term and varying storage conditions may be important limitations affecting data quality from archived samples using proteomics and dSTORM analysis techniques. In addition, variations in sample origin, sex, composition, individual variation and pooling of samples also need to be considered, as well as possible platelet contamination and plasma contaminants, as these may affect both EV recovery and proteomic profiling. It must be emphasised that the pathway annotations for citrullinated candidate proteins in STRING are based on the F95-enriched protein analysis from plasma, using a pan-citrullination IP approach, followed by LC-MS/MS for identification of protein hits, while site-specific mass spectrometry, functional validation and small sample size are limitations to the current study and subject to future investigations. Additional considerations are antibody cross-species reactivity and proteomic reproducibility. Therefore, the discussion is explorative, based on the combined findings from the two archived shark samples used in this study, to establish initial baseline findings.

The aim of the current pilot study was to identify EVs and obtain an overall view of EV proteome cargoes, as well as putative citrullinated protein targets in Greenland shark plasma. This included an assessment of associated pathway annotations for the proteomic analysis. Our results showed that total proteins identified in EV-enriched preparations from Greenland shark plasma were associated with various key metabolic pathways including glycolysis/gluconeogenesis, carbon and purine metabolism, carbon metabolism in cancer, and biosynthesis of amino acids. Immune-related KEGG pathways were associated with proteins identified in EV-enriched preparations linked to inflammation, infection, complement system responses and HIF-1 signalling. These findings indicate roles for EVs in immune and metabolic functions, and in oxidative-stress-related responses. The EV-associated Reactome pathway analysis was linked to innate and adaptive immunity, calcium mobilisation pathways, DNA replication and transcriptional regulation, cellular senescence, cell signalling and metabolism pathways. The EV proteome biological pathway annotation analysis associated with immune system processes and immune defences, acute phase response, iron transport and homeostasis, chemical homeostasis and with various metabolic processes.

For the F95-enriched candidate citrullinated protein hits, in Greenland shark plasma, downstream pathway annotation analysis showed that KEGG pathways were associated with glycolysis/gluconeogenesis, metabolic pathways (including carbon, pyruvate, starch and sucrose metabolism), and biosynthesis of amino acids. Immune-associated KEGG pathways were related to the complement and coagulation cascades, autoimmunity and bacterial infection. These findings were also reflected in the Reactome pathway analysis which showed annotations for various innate and adaptive immune mechanisms, including the complement system, scavenger receptor, and B-cell and NF-κB pathways. Reactome pathway annotations were also associated with fibrin clot and platelet-associated pathways, and cell signalling—including Integrin and MAPK pathways—while metabolic pathways were associated with glycolysis and gluconeogenesis, carbohydrates and IGF transport. Biological pathway annotations were linked to humoral immunity, defence against pathogens and antimicrobial responses, inflammation, acute-phase responses, stress responses and wound healing. Iron transport and homeostasis were also associated with the F95-enriched shark plasma proteins, as well as pathways for regulation of proteolysis and endopeptidase activity. The presence of PAD2 was confirmed in Greenland shark plasma by Western blotting showing positive band detection in the expected 70–75 kDa size range and corresponds with previous reports of PAD2-like protein in nurse shark plasma [26].

Shark immunity is overall well developed with innate and adaptive immune features [7,46,47,48]. The complement system, which plays key roles in immunity, has been studied in several shark species, with reported alternative and classical pathway functions in nurse shark and hammerhead shark [8,9]. Several complement components were identified in the EV-enriched preparation, and included complement components C1, C3, C4, C6, and C8. EV-mediated export of complement components has been reported in several other fish species [25,34,35,36,38,49]. Complement components identified here as candidate citrullinated proteins in the F95-enriched shark plasma preparation were C3 and C4, and C3 was previously reported as a citrullinated protein target in nurse shark plasma [26], as well as in several teleost fish [36,49]. The complement system is key to immune responses against a wide range of pathogens and in this study associations with several immune, including phagocytosis, pathways were identified for the EV-associated proteome and the F95-enriched plasma proteins. Like other sharks and fish, Greenland sharks are exposed to various pathogens. For example, a report on meningitis in a Greenland shark was published in 2023, in the brain and spinal cord with a near-pure growth of a *Vibrio* organism isolated from CSF [50]. High lysozyme activity levels are reported in the Leydig organ, spleen and pancreas of *S. microcephalus* suggesting a well-developed immune response against pathogens [11]. B-cell responses are also of considerable importance, and recent studies have revealed pancreatic B-cell follicles in nurse sharks, with roles in adaptive immune surveillance which may function in the absence of lymph nodes [51]. B-cell-associated pathways were here associated with the citrullinated plasma proteins. An in-depth LC-MS/MS profiling study of the nurse shark plasma immune proteome identified roles for coagulation, including coagulation factors, prothrombin, fibrinogen, plasminogen, several complement components and SERPINs [52], all of which were identified here as candidate citrullinated protein targets in Greenland shark plasma. To what extent post-translational citrullination may contribute to immune functions may be of interest for future investigations.

Secreted IgW heavy chain was identified in the EV-enriched preparations. IgW is part of the adaptive immune responses and is one of the three immunoglobulin isotypes in shark, besides IgM and IgNAR [10,53]. In cartilaginous fish multiple forms of IgW have been described [54]. IgW can perform isotype switching and several secreted isoforms have been identified in nurse shark [55]. Here, secreted IgW heavy chain was found in the enriched EV preparation from Greenland shark plasma and was previously also identified in EVs from nurse shark plasma [26]. Basigin was also identified in the Greenland shark plasma-EV preparations and it is a multifunctional member of the immunoglobulin superfamily, acting as a critical regulator of tumour progression, immune modulation, and metabolic adaptation [56].

Haemoglobin subunit 1 was identified in the EV-enriched preparation from shark plasma, while haemoglobin subunits 1, 2 and 3 were identified as candidate citrullinated hits in the F95-enriched plasma proteins. Three major haemoglobins are reported in shark red blood cells and show very similar oxygenation and carbonylation properties, which are unaffected by urea, which is a very important compound in marine elasmobranch physiology. The haemoglobins of this polar shark have evolved adaptive decreases in O_2_ affinity in comparison to temperate sharks [12]. Haemoglobin was previously reported as a citrullinated protein candidate in nurse shark [26]. It must be considered that the detection of haemoglobin here may possibly be influenced by haemolysis, which cannot be fully excluded in the archived samples used.

Interestingly, the HIF-1 signalling KEGG pathway was associated with proteins in the EV-enriched preparation from shark plasma, and stress response pathways were associated both with the EV- as well as the F95-enriched protein preparations. Oxidative stress has been assessed in Greenland shark and has been suggested to relate to their environmental adaptations to cold water temperatures and deep waters, rather than to longevity [57]. Ceruloplasmin and its homologue hephaestin, which have copper binding functions and ferroxidase activity with roles in cellular iron export [58,59], were candidate hits both in the EV- as well as the F95-enriched plasma proteins. Various other stressors which the sharks are exposed to include pathogens and environmental toxins. As a top predator, the Greenland shark is an ideal sentinel species for ecotoxicological studies and assessment of environmental pollution, with studies assessing heavy metals and trace elements in its tissues [60,61]. This may possibly be related to the various pathway annotations linked to chemical homeostasis and toxins, which were identified for F95-enriched and EV-associated protein hits. Further experimental validation will be needed in future studies.

The unusual longevity of the Greenland shark has gained considerable attention and has partly been attributed to its extreme environment, low metabolic rate and remarkably late sexual maturation, all of which may reduce cumulative physiological stress over time. It has been suggested that additional mechanisms such as proteostatic resilience, antioxidant defences and immune adaptations also support long-term cellular homeostasis in the Greenland shark [14]. Field metabolic rate studies have shown that they require very little energy to sustain themselves under natural conditions [62]. Notably, numerous metabolic pathways were, in our current study, found to be associated with both the EV protein analysis and the F95-enriched plasma protein hits. Mitoguardin 2, a mitochondrial outer membrane protein, was a hit in the EV-enriched fraction. It is reported to play important roles in lipid transfer and maintenance of mitochondrial morphology [63,64], with regulatory roles in lipophagy and autophagy [65]. Mitoguardin also regulates MT fusion, helps maintenance of mtDNA copy numbers and mtDNA integrity [66,67]. Roles for Mitoguardin in anti-tumour innate immunity are also reported [68]. The Greenland shark is considered a model species for negligible senescence [69], and interestingly annotations for cellular senescence pathways were associated with the EV-enriched fraction. At the molecular level, recent genomic studies have identified distinctive features, including duplications of DNA repair genes and structural variation in the tumour suppressor protein p53, which are consistent with enhanced genome maintenance, although their functional significance remains to be experimentally validated [70,71]. Notably, pathway annotations identified here for the EV-enriched fraction included several DNA and RNA regulatory pathways, MAPK signalling and cellular senescence. MAPK pathways were also associated with the F95-enriched plasma proteins. It must though be emphasised that our associations with these pathways are based on in silico bioinformatics annotations and therefore further experimental validation will need to be carried out in future studies.

Interestingly, melanotransferrin, a cell-surface glycoprotein and member of the transferrin superfamily, was identified here in both the EV-enriched fraction and the F95-enriched plasma preparation. In catshark embryos, it is associated with altered iron metabolism responses as part of hypoxia responses [72]. In mammals, lipid-anchored melanotransferrin mediates transferrin-independent iron uptake and has roles in ferritin storage and iron transport across the blood–brain barrier [73]. It is also a significant cancer antigen, elevated in various tumours, and associated with cell proliferation, cell migration and angiogenesis [74]. Melanotransferrin has previously been identified as EV cargo released from melanoma cells [75]. Roles for melanotransferrin have also been reported in neurological disease, associated with the neuroinflammatory response, while its ability to cross the blood–brain barrier have been utilised for developing targeted drug delivery systems [74,76,77]. Studies on the Greenland shark’s distinctive gene repertoire have suggested possible links between its exceptional longevity and ferroptosis pathways [70], which may be of interest as we identified pathway annotations for iron transport and metabolism pathways.

A recent study on gene family expansion and positive selection revealed classical longevity-related mechanisms, including immune enhancement, cancer resistance, and DNA repair, as well as additional features potentially associated with extended lifespan limits. For example, unique amino acid substitutions in the globular domain of linker histone H1.0 are predicted to enhance chromatin stability [70]. A few associations with our findings can be speculated, although this will need further experimental validation. In the current study we identified for example several histones (H2A, H4, Core Histone H2A/H2B/H3) associated with the EV-enriched protein hits, while histones H2A and H4 were F95-enriched plasma protein hits. Resistance to cardiac ageing has furthermore been shown in Greenland shark [78]. Interestingly, Titin was a candidate hit in the F95-enriched plasma proteins. This protein is the largest muscle protein in the body and key to heart mechanistic functions, and a major disease locus for cardiac disorders [79]. Given the low coverage of this large protein in the F95-enriched hit list (Table S4), it must be considered as a tentative target, while putative effects of post-translational citrullination on its functions may be of interest for future studies.

The Greenland shark represents a valuable yet still underexplored species for investigating adaptations to extreme environments and biological processes involved in longevity. While exploratory, our findings provide some new insights relating to EV- and citrullination-associated pathways. These may be of interest for further investigations and contribute to ongoing research on this shark’s immune, metabolic and cell-communication pathways. It must be emphasised that the current study is descriptive and limited by a very small sample size. The pathway annotation analysis in association with EV cargoes and F95-enriched citrullinated candidate proteins is exploratory, including some speculative discussion in relation to the wider literature, and solely based on bioinformatic analysis from the two archived samples. Functional validation, biological replication in other samples and further investigations into age/lifespan comparisons, metabolic processes and immune functions will remain subject to future studies.

## 5. Conclusions

This investigation assessed Greenland shark (*S. microcephalus*) plasma extracellular vesicles (EVs), including profiling by super-resolution microscopy and proteomic content, alongside analysis of enriched citrullinated proteins in two archived shark plasma samples. Explorative proteomic analysis of the EV-enriched plasma fractions was associated with key immune, oxidative stress, DNA replication, cell senescence and metabolic pathways, using explorative bioinformatic approaches. F95-enriched citrullinated plasma protein profiles were associated with pathways relating to gene regulation, stress and immune responses, chemical homeostasis, metabolism and cell signalling. Co-localisation of citrullinated proteins to individual EVs was furthermore confirmed by single-molecule detection using dSTORM imaging. Our findings contribute to the limited knowledge of cellular mechanisms in Greenland shark immunity and physiology. Its unique position as an understudied species for longevity and adaptations to extreme environments may furthermore hold some interest for comparative human physiology and pathobiology studies.

## Figures and Tables

**Table 1:** Details of the two samples (A and B) from Greenland shark (*S. microcephalus*) used in this study. The ID numbers in the UiT The Arctic University of Norway sample database of Greenland shark from the Northern Atlantic are included alongside area of catch, sex, and total length (TL) in cm. Note that sample A was obtained from one individual caught in the TUNU IV expedition, while sample B is a pool from three sharks caught during the TUNU VI expedition.

Sample	ID	Expedition	Date	Area	Location	Position	Depth (m)	Sex	TL (cm)	Storage Temp.
**A**	GS164	TUNU IV	13.08.2010	NE Greenland	Kejser Franz Joseph Fjord	73°26′34.4″ N, 23°48′07.3″ W	473	F	330	−20 °C
**B** **(Pool of 3 sharks)**	GS181	TUNU VI	11.08.2015	NE Greenland	NE Greenland shelf	75°04′00.0″ N, 12°42′00.0″ W	377	M	271	−80 °C
	GS182	TUNU VI	11.08.2015	NE Greenland	NE Greenland shelf	75°04′00.0″ N, 12°42′00.0″ W	377	F	322	
	GS183	TUNU VI	11.08.2015	NE Greenland	NE Greenland shelf	75°04′00.0″ N, 12°42′00.0″ W	377	F	320	

**Table 2:** Protein hits identified in the EV-enriched preparations from Greenland shark plasma (samples A and B) by LC-MS/MS. Protein ID, protein name, species and hit score are presented. Individual ion scores > 37 indicate identity or extensive homology (*p* < 0.05). Protein scores are derived from ion scores as a non-probabilistic basis for ranking protein hits; the search was conducted using the UniProt Chondrichthyes database Chondrichthyes_180526 (142,694 sequences; 65,411,590 residues).

Protein ID	Protein Name	Species	Common Name	Score A/B
C0HJZ3 C0HJZ2	Haemoglobin subunit beta-1	*Somniosus microcephalus*	Greenland shark	326/393 240/308
A0A401PJX3 A0A401SIL8	Vitellogenin domain-containing protein	*Scyliorhinus torazame* *Chiloscyllium punctatum*	Cloudy catshark Brownbanded bamboo shark	291/122 136/0
A0A401NSU0	Inter-alpha-trypsin inhibitor heavy chain H2	*Scyliorhinus torazame*	Cloudy catshark	0/46
A0A401NGS8 A0A401S592	Inter-alpha-trypsin inhibitor heavy chain H3	*Scyliorhinus torazame* *Chiloscyllium punctatum*	Cloudy catshark Brownbanded bamboo shark	164/252 0/252
H9LDW9	Complement protein 1S	*Squalus acanthias*	Spiny dogfish	56/137
A0A088MN23	C3 complement component	*Ginglymostoma cirratum*	Nurse shark	62/90
A0A146GEJ4	C3A	*Sphyrna zygaena*	Smooth hammerhead	49/126
A0A1J1DSR1	Complement C4 isoform-B	*Triakis scyllium*	Banded houndshark	145/236
A0A1J1E4M7	Complement C4 isoform-C	*Triakis scyllium*	Banded houndshark	0/155
Q76KI4	Complement C4	*Triakis scyllium*	Banded houndshark	0/128
C7S852	Complement component 5 protein	*Ginglymostoma cirratum*	Nurse shark	85/137
A0A146GEV4	C6	*Sphyrna zygaena*	Smooth hammerhead	52/0
A0A146GEW5	C8a	*Sphyrna zygaena*	Smooth hammerhead	54/0
A0A401PBK1	Fibrinogen C-terminal domain-containing protein	*Scyliorhinus torazame*	Cloudy catshark	128/127
K4FUN8	Fibrinogen beta chain	*Callorhinchus milii*	Australian ghostshark	0/91
A0A4W3HYI8	Apolipoprotein B	*Callorhinchus milii*	Australian ghostshark	107/140
A0A401P2Q1 A0A4W3I1Y5	Ceruloplasmin	*Scyliorhinus torazame* *Callorhinchus milii*	Cloudy catshark Australian ghostshark	106/91 57/0
A0A401P0B8 Q6WP72	Glyceraldehyde-3-phosphate dehydrogenase	*Scyliorhinus torazame* *Hypanus sabinus*	Cloudy catshark Atlantic stingray	106/238 0/233
P00341	L-lactate dehydrogenase A chain	*Squalus acanthias*	Spiny dogfish	99/354
P23085	Ig heavy chain C region	*Heterodontus francisci*	Horn shark	182/174
A0A401P9R7 A0A401SE28 A0A401NZ53 A0A4W3GB25	Ig-like domain-containing protein	*Scyliorhinus torazame* *Chiloscyllium punctatum* *Scyliorhinus torazame* *Callorhinchus milii*	Cloudy catshark Brownbanded bamboo shark Cloudy catshark Australian ghostshark	94/0 75/76 71/71 40/41
A0A401SC79	Melanotransferrin	*Chiloscyllium punctatum*	Brownbanded bamboo shark	80/78
A0A146GEN9	C43	*Sphyrna zygaena*	Smooth hammerhead	79/108
A0A401T7V2	Hephaestin	*Chiloscyllium punctatum*	Brownbanded bamboo shark	78/37
A0A401PAM1	NTR domain-containing protein	*Scyliorhinus torazame*	Cloudy catshark	77/79
A0A401RZK4 A0A401RZK0	Transferrin-like domain-containing protein	*Chiloscyllium punctatum*	Brownbanded bamboo shark	62/60 0/80
A0A401RPC7 V9KFX0	Alpha-1,4 glucan phosphorylase	*Chiloscyllium punctatum* *Callorhinchus milii*	Brownbanded bamboo shark Australian ghostshark	56/469 0/442
A0A401QNP3	Glycogen phosphorylase	*Scyliorhinus torazame*	Cloudy catshark	53/224
A0A401RF39	Lipocalin/cytosolic fatty-acid binding domain-containing protein	*Chiloscyllium punctatum*	Brownbanded bamboo shark	51/0
A0A401PRH5	Protein FAM221B	*Scyliorhinus torazame*	Cloudy catshark	49/0
A0A401Q4Y2	Sushi domain-containing protein	*Scyliorhinus torazame*	Cloudy catshark	48/0
A0A401RHK4	Ubiquitin carboxyl-terminal hydrolase	*Chiloscyllium punctatum*	Brownbanded bamboo shark	47/0
A0A4W3IN01 A0A401T753	Anaphylatoxin-like domain-containing protein	*Callorhinchus milii* *Chiloscyllium punctatum*	Australian ghostshark Brownbanded bamboo shark	47/73 41/147
A0A401STM5	RING-type E3 ubiquitin transferase	*Chiloscyllium punctatum*	Brownbanded bamboo shark	47/0
A0A401S6V4	DNA oxidative demethylase ALKBH2	*Chiloscyllium punctatum*	Brownbanded bamboo shark	47/42
A0A401NVR3 A0A401SAN7	Thyroxine-binding globulin	*Scyliorhinus torazame* *Chiloscyllium punctatum*	Cloudy catshark Brownbanded bamboo shark	46/0 0/117
A0A401SAS6	Serpin domain-containing protein	*Chiloscyllium punctatum*	Brownbanded bamboo shark	46/42
A0A401P8J3 A0A401RQ96	Fibronectin	*Scyliorhinus torazame* *Chiloscyllium punctatum*	Cloudy catshark Brownbanded bamboo shark	45/0 40/40
A0A4W3IUB5	Glutathione S-transferase rho	*Callorhinchus milii*	Australian ghostshark	44/0
A0A401SWP5	Rap-GAP domain-containing protein	*Chiloscyllium punctatum*	Brownbanded bamboo shark	40/0
A0A4W3HM19	TBC domain-containing protein kinase-like protein	*Callorhinchus milii*	Australian ghostshark	39/0
A0A4W3I5H5	Glutamate receptor	*Callorhinchus milii*	Australian ghostshark	39/0
A0A401S357	CARD domain-containing protein	*Chiloscyllium punctatum*	Brownbanded bamboo shark	39/0
A0A401SBV6 A0A401PGK1 A0A4W3IA17	Pyruvate kinase	*Chiloscyllium punctatum* *Scyliorhinus torazame* *Callorhinchus milii*	Brownbanded bamboo shark Cloudy catshark Australian ghostshark	0/309 0/301 0/178
Q76BC5 Q76BB8 V9KW23	Fructose-bisphosphate aldolase	*Cephaloscyllium umbratile* *Potamotrygon motoro* *Callorhinchus milii*	Blotchy swellshark Ocellate river stingray Australian ghostshark	0/308 0/153 0/138
A0A292GEA1 I0J0X5	Beta actin	*Scyliorhinus torazame* *Hemitrygon akajei*	Cloudy catshark Red stingray	0/264 0/236
P04414 A0A4W3H1J1 A0A401RNS5 A0A401RNS5 A0A401PYC2	Creatine kinase M-type	*Tetronarce californica* *Callorhinchus milii* *Chiloscyllium punctatum* *Scyliorhinus torazame*	Pacific electric ray Australian ghostshark Brownbanded bamboo shark Cloudy catshark	0/255 0/222 0/181 0/93
A0A401NVB2	ACTC protein	*Scyliorhinus torazame*	Cloudy catshark	0/233
A0A401NUU0 A0A401T4Q7	Phosphoglucomutase-1	*Scyliorhinus torazame* *Chiloscyllium punctatum*	Cloudy catshark Brownbanded bamboo shark	0/217 0/111
A0A401RPB9 A0A401NNU5	Histone H2A	*Chiloscyllium punctatum* *Scyliorhinus torazame*	Brownbanded bamboo shark Cloudy catshark	0/161 0/84
A0A401RTT7	Core Histone H2A/H2B/H3 domain-containing protein	*Chiloscyllium punctatum*	Brownbanded bamboo shark	0/146
A0A401Q4C7	Histone H4	*Scyliorhinus torazame*	Cloudy catshark	0/314
V9KVQ5 Q9DDG8	Beta-enolase	*Callorhinchus milii* *Chiloscyllium punctatum*	Australian ghostshark Brownbanded bamboo shark	0/149 0/114
A0A401T746 A0A401NV46	AMP deaminase	*Chiloscyllium punctatum* *Scyliorhinus torazame*	Brownbanded bamboo shark Cloudy catshark	0/147 0/100
A0A401RPM3	Glucose-6-phosphate isomerase	*Chiloscyllium punctatum*	Brownbanded bamboo shark	0/146
V9KG15	ATP-dependent 6-phosphofructokinase	*Callorhinchus milii*	Australian ghostshark	0/132
Q9YI35 K4FY57	Nucleoside diphosphate kinase	*Scyliorhinus torazame* *Callorhinchus milii*	Cloudy catshark Australian ghostshark	0/121
V9KUX9 Q76BB2	Triosephosphate isomerase	*Callorhinchus milii*	Australian ghostshark Ocellate river stingray	0/100
A0A401SZS6	Adenylate kinase isoenzyme 1	*Chiloscyllium punctatum*	Brownbanded bamboo shark	0/75
U5NFT0	Secreted IgW heavy chain	*Ginglymostoma cirratum*	Nurse shark	0/67
A0A401SL10	Annexin	*Chiloscyllium punctatum*	Brownbanded bamboo shark	0/65
A0A401S5P6	Sodium/potassium/calcium exchanger 5	*Chiloscyllium punctatum*	Brownbanded bamboo shark	0/64
A0A401PLW3	Phosphoglycerate kinase	*Scyliorhinus torazame*	Cloudy catshark	0/57
A0A401Q2P6	Proteasome subunit beta	*Scyliorhinus torazame*	Cloudy catshark	0/50
A0A401PEC6	Multidrug and toxin extrusion protein	*Scyliorhinus torazame*	Cloudy catshark	0/49
A0A401RSD6	ELK domain-containing protein	*Chiloscyllium punctatum*	Brownbanded bamboo shark	0/48
A0A401NI68	Ubiquitin-ribosomal protein eS31 fusion protein	*Scyliorhinus torazame*	Cloudy catshark	0/47
A0A401SL78	PWWP domain-containing protein	*Chiloscyllium punctatum*	Brownbanded bamboo shark	0/45
A0A401NXE9	Mitoguardin 2	*Scyliorhinus torazame*	Cloudy catshark	0/43
A0A401Q019	Peptidase S1 domain-containing protein	*Scyliorhinus torazame*	Cloudy catshark	0/41
A0A4W3HGP1	Adenylate cyclase type 2	*Callorhinchus milii*	Australian ghostshark	0/40
A0A401TJ77	R domain-containing protein	*Chiloscyllium punctatum*	Brownbanded bamboo shark	0/40
V9KZF6	Cilia- and flagella-associated protein 45	*Callorhinchus milii*	Australian ghostshark	0/40
A0A401SUT2	Glycogen debranching enzyme	*Chiloscyllium punctatum*	Brownbanded bamboo shark	0/39
A0A401NNQ2	Basigin	*Scyliorhinus torazame*	Cloudy catshark	0/39
A0A4W3IN79	F-box protein 16	*Callorhinchus milii*	Australian ghostshark	0/38
A0A401T7S6	Uncharacterised protein	*Chiloscyllium punctatum*	Brownbanded bamboo shark	0/38
A0A401SFU7	Uncharacterised protein	*Chiloscyllium punctatum*	Brownbanded bamboo shark	84/132
A0A401PJI5	Uncharacterised protein	*Scyliorhinus torazame*	Cloudy catshark	83/217
A0A401PJV2	Uncharacterised protein	*Scyliorhinus torazame*	Cloudy catshark	70/136
A0A401T793	Uncharacterised protein	*Chiloscyllium punctatum*	Brownbanded bamboo shark	0/153
A0A401SG04	Uncharacterised protein	*Chiloscyllium punctatum*	Brownbanded bamboo shark	0/97
A0A401TLB2	Uncharacterised protein	*Chiloscyllium punctatum*	Brownbanded bamboo shark	0/50

**Table 3:** F95-enriched candidate citrullinated protein hits identified in Greenland shark plasma by LC-MS/MS in samples A and B. Protein ID, protein name, species and hit score are presented. Individual ion scores > 36 indicate identity or extensive homology (*p* < 0.05). Protein scores are derived from ion scores as a non-probabilistic basis for ranking protein hits; the search was conducted using the UniProt Chondrichthyes database Chondrichthyes_180526 (142,694 sequences; 65,411,590 residues).

Protein ID	Protein Name	Species	Common Name	Score A/B
C0HJZ3	Haemoglobin subunit beta-1	*Somniosus microcephalus*	Greenland shark	199/515
C0HJZ4	Haemoglobin subunit beta-2	*Somniosus microcephalus*	Greenland shark	199/629
C0HJZ5	Haemoglobin subunit beta-3	*Somniosus microcephalus*	Greenland shark	199/620
P07409	Haemoglobin subunit beta	*Squalus acanthias*	Spiny dogfish	90/212
C0HJZ2	Haemoglobin subunit alpha	*Somniosus microcephalus*	Greenland shark	176/366
K4FUN8	Fibrinogen beta chain	*Callorhinchus milii*	Australian ghostshark	117/270
A0A401PBK1	Fibrinogen C-terminal domain-containing protein	*Scyliorhinus torazame*	Cloudy catshark	78/232
A0A401Q6K6	Olfactomedin-like domain-containing protein	*Scyliorhinus torazame*	Cloudy catshark	83/77
P23085	Ig heavy chain C region	*Heterodontus francisci*	Horn shark	191/208
A0A401P9R7 A0A4W3GB25 A0A401NZ53 A0A401SE28	Ig-like domain-containing protein	*Scyliorhinus torazame* *Callorhinchus milii* *Scyliorhinus torazame* *Chiloscyllium punctatum*	Cloudy catshark Australian ghostshark Cloudy catshark Brownbanded bamboo shark	71/42 66/40 59/69 41/108
A0A401SC79	Melanotransferrin	*Chiloscyllium punctatum*	Brownbanded bamboo shark	61/0
A0A401RZK4	Transferrin-like domain-containing protein	*Chiloscyllium punctatum*	Brownbanded bamboo shark	58/57
A0A401PAM1	NTR domain-containing protein	*Scyliorhinus torazame*	Cloudy catshark	44/0
A0A401PRH5	Protein FAM221B	*Scyliorhinus torazame*	Cloudy catshark	43/0
A0A401Q019	Peptidase S1 domain-containing protein	*Scyliorhinus torazame*	Cloudy catshark	42/45
A0A401RNS5 A0A4W3H1J1 A0A401RNS5 A0A401P7F5	Creatine kinase M-type	*Chiloscyllium punctatum* *Callorhinchus milii* *Chiloscyllium punctatum* *Scyliorhinus torazame*	Brownbanded bamboo shark Australian ghostshark Brownbanded bamboo shark Cloudy catshark	37/123 0/177 0/123 0/78
Q76BB3	Phosphoglycerate kinase	*Potamotrygon motoro*	Ocellate river stingray	0/77
A0A401SBV6	Pyruvate kinase	*Chiloscyllium punctatum*	Brownbanded bamboo shark	0/208
A0A401NGS8	Inter-alpha-trypsin inhibitor heavy chain H3	*Scyliorhinus torazame*	Cloudy catshark	0/291
A0A401P2Q1	Ceruloplasmin	*Scyliorhinus torazame*	Cloudy catshark	0/245
A0A401P0B8 Q6WP72	Glyceraldehyde-3-phosphate dehydrogenase	*Scyliorhinus torazame* *Hypanus sabinus*	Cloudy catshark Atlantic stingray	0/220 0/218
A0A401RPC7	Alpha-1,4 glucan phosphorylase	*Chiloscyllium punctatum*	Brownbanded bamboo shark	0/205
P00341	L-lactate dehydrogenase A	*Squalus acanthias*	Spiny dogfish	0/199
Q76BC5 Q76BB8 V9KW23	Fructose-bisphosphate aldolase	*Cephaloscyllium umbratile* *Potamotrygon motoro* *Callorhinchus milii*	Blotchy swellshark Ocellate river stingray Australian ghostshark	0/189 0/150 0/127
A0A401RQ96 A0A401P8J3	Fibronectin	*Chiloscyllium punctatum* *Scyliorhinus torazame*	Brownbanded bamboo shark Cloudy catshark	0/171 0/129
A0A4W3HYI8	Apolipoprotein B	*Callorhinchus milii*	Australian ghostshark	0/145
A0A401T4Q7 A0A401NUU	Phosphoglucomutase-1	*Chiloscyllium punctatum* *Scyliorhinus torazame*	Brownbanded bamboo shark Cloudy catshark	0/144 0/132
Q9YI35	Nucleoside diphosphate kinase	*Scyliorhinus torazame*	Cloudy catshark	0/137
A0A401T7V	Hephaestin	*Chiloscyllium punctatum*	Brownbanded bamboo shark	0/130
A0A292GEA1	Beta actin	*Scyliorhinus torazame*	Cloudy catshark	0/123
A0A401NVB2	ACTC protein	*Scyliorhinus torazame*	Cloudy catshark	0/115
A0A401RPB9	Histone H2A	*Chiloscyllium punctatum*	Brownbanded bamboo shark	0/88
A0A401Q4C7	Histone H4	*Scyliorhinus torazame*	Cloudy catshark	0/113
A0A401PJX3	Vitellogenin	*Scyliorhinus torazame*	Cloudy catshark	0/109
A0A401RPM3	Glucose-6-phosphate isomerase	*Chiloscyllium punctatum*	Brownbanded bamboo shark	0/98
A0A401RF17	Noelin domain-containing protein	*Chiloscyllium punctatum*	Brownbanded bamboo shark	0/98
V9KUX9	Triosephosphate isomerase	*Callorhinchus milii*	Australian ghostshark	0/88
A0A4W3J4G6	Complement C1q tumour necrosis factor-related protein 4	*Callorhinchus milii*	Australian ghostshark	0/40
A0A088MN23	C3 complement component	*Ginglymostoma cirratum*	Nurse shark	0/67
A0A146GEJ4	C3A	*Sphyrna zygaena*	Smooth hammerhead	0/86
C7S852	Complement component 5 protein	*Ginglymostoma cirratum*	Nurse shark	0/54
A0A401T753	Anaphylatoxin-like domain-containing protein	*Chiloscyllium punctatum*	Brownbanded bamboo shark	0/86
A0A4W3HAL5	6-phosphofructokinase	*Callorhinchus milii*	Australian ghostshark	0/75
A0A4W3H187	Alpha-1,4 glucan phosphorylase	*Callorhinchus milii*	Australian ghostshark	0/74
Q76BB2	Triosephosphate isomerase	*Potamotrygon motoro*	Ocellate river stingray	0/70
A0A401T4Q3	Prothrombin	*Chiloscyllium punctatum*	Brownbanded bamboo shark	0/69
A0A401QNP3	Glycogen phosphorylase	*Scyliorhinus torazame*	Cloudy catshark	0/65
A0A4W3GJH7	Ral GTPase activating protein catalytic subunit alpha 1	*Callorhinchus milii*	Australian ghostshark	0/63
K4FY57	Nucleoside diphosphate kinase	*Callorhinchus milii*	Australian ghostshark	0/60
V9KVQ5	Beta-enolase	*Callorhinchus milii*	Australian ghostshark	0/59
D0PQG3	Col1a1	*Squalus acanthias*	Spiny dogfish	0/56
A0A401NGR7	IF rod domain-containing protein	*Scyliorhinus torazame*	Cloudy catshark	0/54
A0A401S8F4	Plasminogen	*Chiloscyllium punctatum*	Brownbanded bamboo shark	0/52
V9K7X8	Golgin subfamily B member 1-like protein	*Callorhinchus milii*	Australian ghostshark	0/51
A0A401S3B8	Aryl hydrocarbon receptor	*Chiloscyllium punctatum*	Brownbanded bamboo shark	0/51
A0A401T2L0	Non-specific serine/threonine protein kinase	*Chiloscyllium punctatum*	Brownbanded bamboo shark	0/50
A0A401RSD6	ELK domain-containing protein	*Chiloscyllium punctatum*	Brownbanded bamboo shark	0/50
A0A401Q4Y2	Sushi domain-containing protein	*Scyliorhinus torazame*	Cloudy catshark	0/47
A0A401PBK3	Fibrinogen alpha/beta/gamma chain coiled coil domain-containing protein	*Scyliorhinus torazame*	Cloudy catshark	0/47
A0A401RZC2	ATP-grasp domain-containing protein	*Chiloscyllium punctatum*	Brownbanded bamboo shark	0/46
A0A401P3E2	Titin	*Scyliorhinus torazame*	Cloudy catshark	0/45
A0A0H4IQM0	Antithrombin-III	*Chiloscyllium punctatum*	Brownbanded bamboo shark	0/43
A0A401SAF0	Protein Hook homologue 3	*Chiloscyllium punctatum*	Brownbanded bamboo shark	0/42
A0A401SA64	Ferroxidase	*Chiloscyllium punctatum*	Brownbanded bamboo shark	0/42
A0A401NP72	Glycerol-3-phosphate dehydrogenase [NAD(+)]	*Scyliorhinus torazame*	Cloudy catshark	0/42
A0A401T6C4	WW domain-containing protein	*Chiloscyllium punctatum*	Brownbanded bamboo shark	0/41
A0A401S5T7	Alanine transaminase	*Chiloscyllium punctatum*	Brownbanded bamboo shark	0/40
A0A401PQS5	Glycogen debranching enzyme	*Scyliorhinus torazame*	Cloudy catshark	0/39
A0A401PCD2	DUF4585 domain-containing protein	*Scyliorhinus torazame*	Cloudy catshark	0/39
A0A401NVR3	Thyroxine-binding globulin	*Scyliorhinus torazame*	Cloudy catshark	0/39
A0A401PE57	Collagen alpha-1(I) chain	*Scyliorhinus torazame*	Cloudy catshark	0/39
A0A146GEN9	C43	*Sphyrna zygaena*	Smooth hammerhead	0/39
A0A401Q056	Serpin domain-containing protein	*Scyliorhinus torazame*	Cloudy catshark	0/38
A0A401PIT3	HTH CENPB-type domain-containing protein	*Scyliorhinus torazame*	Cloudy catshark	0/37
A0A4W3J5H8	Non-specific serine/threonine protein kinase	*Callorhinchus milii*	Australian ghostshark	0/36
A0A146GFD3	CLP13	*Sphyrna zygaena*	Smooth hammerhead	0/36
A0A401SFU7	Uncharacterised protein	*Chiloscyllium punctatum*	Brownbanded bamboo shark	50/151
A0A401PJV2	Uncharacterised protein	*Scyliorhinus torazame*	Cloudy catshark	0/74
A0A401T7S6	Uncharacterised protein	*Chiloscyllium punctatum*	Brownbanded bamboo shark	0/38
A0A401QID6	Uncharacterised protein	*Scyliorhinus torazame*	Cloudy catshark	0/41
A0A401T793	Uncharacterised protein	*Chiloscyllium punctatum*	Brownbanded bamboo shark	0/56
A0A401U3E9	Uncharacterised protein	*Sphyrna zygaena*	Smooth hammerhead	0/45

## Data Availability

The original contributions presented in this study are included in the article/Supplementary Material. Further inquiries can be directed to the corresponding author.

## Supplementary Materials

The following supporting information can be downloaded at https://www.mdpi.com/article/10.3390/biology15201867/s1.
